# Enhanced Metabolic Potentials and Functional Gene Interactions of Microbial Stress Responses to a 4,100-m Elevational Increase in Freshwater Lakes

**DOI:** 10.3389/fmicb.2020.595967

**Published:** 2021-01-13

**Authors:** Huabing Li, Jin Zeng, Lijuan Ren, Qingyun Yan, Qinglong L. Wu

**Affiliations:** ^1^State Key Laboratory of Lake Science and Environment, Nanjing Institute of Geography and Limnology, Chinese Academy of Sciences, Nanjing, China; ^2^Department of Ecology and Institute of Hydrobiology, Jinan University, Guangzhou, China; ^3^Environmental Microbiomics Research Center, School of Environmental Science and Engineering, Southern Marine Science and Engineering Guangdong Laboratory (Zhuhai), Sun Yat-sen University, Guangzhou, China; ^4^Sino-Danish Centre for Education and Research, University of Chinese Academy of Sciences, Beijing, China

**Keywords:** elevation, freshwater lakes, microbial functional genes, stress response, metabolic potentials

## Abstract

Elevation has a strong influence on microbial community composition, but its influence on microbial functional genes remains unclear in the aquatic ecosystem. In this study, the functional gene structure of microbes in two lakes at low elevation (ca. 530 m) and two lakes at high elevation (ca. 4,600 m) was examined using a comprehensive functional gene array GeoChip 5.0. Microbial functional composition, but not functional gene richness, was significantly different between the low- and high-elevation lakes. The greatest difference was that microbial communities from high-elevation lakes were enriched in functional genes of stress responses, including cold shock, oxygen limitation, osmotic stress, nitrogen limitation, phosphate limitation, glucose limitation, radiation stress, heat shock, protein stress, and sigma factor genes compared with microbial communities from the low-elevation lakes. Higher metabolic potentials were also observed in the degradation of aromatic compounds, chitin, cellulose, and hemicellulose at higher elevations. Only one phytate degradation gene and one nitrate reduction gene were enriched in the high-elevation lakes. Furthermore, the enhanced interactions and complexity among the co-occurring functional genes in microbial communities of lakes at high elevations were revealed in terms of network size, links, connectivity, and clustering coefficients, and there were more functional genes of stress responses mediating the module hub of this network. The findings of this study highlight the well-developed functional strategies utilized by aquatic microbial communities to withstand the harsh conditions at high elevations.

## Introduction

Elevational patterns of biodiversity have attracted interest in the scientific fields of microbial ecology and biogeography because of the importance of these patterns in facilitating a comprehensive understanding of the influences of climate change on ecosystems. Numerous studies concerning elevational gradients have focused on terrestrial organisms and revealed that species richness generally exhibits decreasing or unimodal patterns with increases in elevation (e.g., Rahbek, 2005; Grytnes and Mccain, 2007). Studies on elevational species richness of a few freshwater taxa including aquatic plants (Jones et al., 2003), phytoplankton (Jankowski and Weyhenmeyer, 2006), rotifers (Obertegger et al., 2010), crustaceans (Hessen et al., 2007), stream macro-invertebrates (Jacobsen, 2004), and molluscs (Sturm, 2007) reported a linear decreasing pattern in species richness with elevation. In contrast, studies on chironomids (Nyman et al., 2005) and stream fish (Bhatt et al., 2012) showed a hump-shaped pattern in species richness with elevation. However, studies focusing on microbial diversity across elevation gradients in freshwater lakes are limited (Hayden and Beman, 2016; Li et al., 2017), even though microbial communities are the most important groups driving biogeochemical cycles and sustaining the whole ecosystem in lakes (Cole et al., 2000).

Microbial communities play central roles in biogeochemical processes such as carbon, nitrogen, and phosphorus cycling (Whitman et al., 1998; Cole et al., 2000). The development of microbial molecular biology technologies has led to considerable attention being directed at the phylogenetic and functional diversity patterns of microbial communities along elevations (Wang et al., 2011; Hayden and Beman, 2016; Li et al., 2017). However, most of these studies focused on taxonomy. The limited number of studies on microbial communities inhabiting soils (Bryant et al., 2008; Fierer et al., 2011; Singh et al., 2012), stones in streams (Wang et al., 2011), leaf surfaces (Fierer et al., 2011), and lake waters (Hayden and Beman, 2016; Li et al., 2017) indicated that the elevational patterns of microbial species richness varied with elevational ranges or habitat types. Microbial functional metabolic potentials are influenced by the changes in microbial species richness and environmental conditions (Kuang et al., 2016). However, little is known about the elevational patterns of microbial functional traits. Elucidating these patterns could improve our understanding of the influences of climate change on microbial-related ecological processes.

Many of the environmental factors that vary with elevation might affect the functional gene structure of microbial communities. Studies on microbial functional gene diversity along elevation gradients are very limited (Yang et al., 2014; Qi et al., 2017; Picazo et al., 2020). Yang et al. (2014) and Qi et al. (2017) reported that soil microbial functional gene structure (MFS) changed significantly with an increase in elevation from 3,200 to 3,800 m, and these changes were predominantly caused by decreases in temperature and concentrations of nutrients. A similar pattern was observed in biofilm microbes along three elevational gradients lower than 4,000 m (Picazo et al., 2020). The shifts in these environmental variables in lakes can be greater than twofold between elevations of approximately 530 to 4,600 m (Li et al., 2017). Furthermore, in contrast to data obtained from elevational studies of grassland, the high-elevation lakes (HELs), especially those with elevations above 4,000 m, are characterized by harsher conditions, including high UV, low primary production, and more recalcitrant dissolved organic carbon (DOC), than those of the low-elevation lakes (LELs; Li et al., 2017; Zhou et al., 2018). The harsh conditions in HELs are hypothesized to result in distinct patterns of microbial metabolic potentials compared to those in LELs, but this has not yet been investigated. It has been confirmed that microbial recruitment to exposed rocky surface habitats is based strongly on the selection of stress tolerance traits (Chan et al., 2013). It is thus very likely that microbial communities of HELs might have higher functional capacities in stress responses such as cold shock and radiation stress, and higher metabolic potentials in the degradation of recalcitrant DOC.

In an ecosystem, microorganisms interact with other organisms and their environment to form a complicated network (Zengler and Zaramela, 2018). Understanding this complex network over time and space is a key issue in ecology (Montoya et al., 2006; Zengler and Zaramela, 2018). Network analysis has been proven as a powerful method for examining complex interactions among microbes and identifying keystone species in different ecosystems, such as the human gut (Greenblum et al., 2012), river (Hu et al., 2017), marine environment (Gilbert et al., 2012), soils (Zhou et al., 2011; Barberán et al., 2012), and rhizosphere (Shi et al., 2016). Furthermore, this method has been applied to investigate changes in microbial metabolic potentials in elevated atmospheric CO_2_ and oil-contaminated soils (Zhou et al., 2010; Liang et al., 2011) and an acidic mining lake (Ren et al., 2018). These studies provide perspectives on microbial assemblages that cannot easily be found by simple alpha- and beta-diversity analyses (Shi et al., 2016). The network complexity of different microbial functional genes under elevated atmospheric CO_2_ or more stress conditions (e.g., low pH, limited carbon, nitrogen, and phosphorus sources) increased in terms of network size, connectivity, and clustering coefficients (Zhou et al., 2010; Ren et al., 2018). However, the patterns of microbial functional gene interactions have not yet been investigated in lakes with contrasting patterns of environmental variables at different elevations.

In this study, it was hypothesized that (1) the MFS differs significantly among lakes under different elevations; (2) the microbial functional potentials of stress responses increase with harsher environments at high elevations; (3) the harsh conditions of HELs alter the microbial functional molecular ecological network. These hypotheses were tested by focusing on four shallow freshwater lakes, including two HELs (at 4,652 and 4,608 m) and two LELs (at 530 and 525 m), at Mount Siguniang in Sichuan province, China. A comprehensive functional gene array (GeoChip 5.0) was utilized to analyze the functional gene diversity and metabolic potentials of the microbial communities in the four lakes. Random matrix theory (RMT)-based network analysis was performed to compare the interaction patterns of the co-occurring functional genes between the HELs and LELs. Overall, the contrasting patterns of freshwater microbial metabolic potentials and functional gene interactions between the HELs and LELs were demonstrated.

## Materials and Methods

### Field Sampling and Measurement of Environmental Parameters

The sampling sites were two HELs and two LELs at Mount Siguniang, a mountain system located in the eastern region of the Tibetan Plateau in China. Mount Siguniang, often referred to as the “Alps of Asia,” comprises a number of peaks, the highest of which has an elevation of 6,250 m. The upper slopes of the high mountains are mostly covered by snow throughout the year, which offers a desirable elevation gradient as well as a relatively geologically uniform environmental gradient within a small spatial scale (Li et al., 2017). The elevations of the two LELs were 525 and 530 m above sea level, whereas those of the two HELs were 4,652 and 4,608 m above sea level (Li et al., 2017). The two LELs (Maojiakou and Baigongyan; Supplementary Table 1 and Supplementary Figure 1) had much higher water temperature and concentrations of DOC, Chl *a*, and nutrients compared with the two HELs (Heihaizi and Baihaizi; Supplementary Table 1 and Supplementary Figure 1). Morphometric variables (elevation, latitude, longitude, surface area, and maximum depth) of each lake were measured and six uniformly distributed sites were selected in each lake for sampling. At each sampling point, approximately 1 and 9 L of surface water (top 50 cm) were collected for analysis of microbial functional structure and chemical variables, respectively. The methods used for sampling and determination of environmental characteristics were as described by Li et al. (2017).

### DNA Extraction and GeoChip Analysis

Total microbial nucleic acids were extracted and purified as described previously (Li et al., 2017). GeoChip 5.0, which includes more than 57,000 oligonucleotide probes and covers over 144,000 gene sequences from 393 functional gene families involved in cycling of carbon, nitrogen, phosphorus, and sulfur, stress response, etc. (Wang et al., 2014), was used to analyze the obtained DNA samples. For each sample, the DNA (500 ng) was labeled with Cy-3 fluorescent dye (GE Healthcare, CA, United States) by random priming as described previously (He et al., 2010) and then the labeled DNA was purified using a QIAquick purification kit (Qiagen, CA, United States) and dried in a SpeedVac (Thermo Savant, NY, United States). Dried DNA was resuspended in 42 μl of hybridization solution containing 1 × Hi-RPM hybridization buffer, 1 × CGH blocking agent, 0.05 μg μl^–1^ Cot-1 DNA, 10 pM universal standard, and 10% formamide (final concentrations). After mixing completely, the mixture was denatured at 95°C for 3 min and then kept at 37°C until hybridization. The hybridization was conducted at 67°C in an Agilent hybridization station (Agilent Technologies Inc., Santa Clara, CA, United States). Hybridized GeoChips were scanned by a NimbleGen MS200 scanner (Roche NimbleGen, Inc., Madison, WI, United States) and the image data were extracted using Agilent Feature Extraction 11.5 software (Agilent Technologies).

### Data Preprocessing

Raw data were uploaded to the microarray analysis pipeline^1^ and analyzed as previously described (Yang et al., 2014; Yan et al., 2015). Briefly, the following steps were performed: (i) good quality spots were selected, which were defined as having a signal-to-noise ratio (SNR) > 2; (ii) probes that appeared in more than approximately 30% of samples in one lake (two of the six samples from each lake) were selected for subsequent analysis; (iii) for each sample, the signal intensity of each probe was ln (*x*+1)-transformed and then normalized by dividing the initial signal intensity of each probe by the mean intensity of positive probes in each sample (Chan et al., 2013). The obtained microarray data matrix (normalized signal intensity) was considered as “species” abundance for microbial functional gene richness and composition analysis (Zhou et al., 2010); and (iv) the normalized signal intensities of all spots of each sample were transferred into relative signal intensities by dividing the normalized signal intensity of a probe by the total normalized signal intensity of a sample. These relative signal intensities were used for further evaluation of the microbial metabolic potentials (Ren et al., 2018). Finally, (v) the difference in normalized signal intensity (or the difference in relative signal intensity) of a specific gene family was calculated as: (S_HELs_/S_LELs_) - 1, where the S_HELs_ and S_LELs_ are the total normalized signal intensity of a specific gene family in the HELs and LELs, respectively.

### Network Construction

To reduce the complexity of the datasets, this study specifically focused on the commonly detected functional genes involved in key biogeochemical and ecological processes in lakes along elevation gradients, including carbon, nitrogen, and phosphorus cycling, and stress responses. These genes in the LELs and HELs were selected for the construction of the functional molecular ecological networks (fMENs), which were based on a RMT approach using a publicly available Molecular Ecological Network Analysis Pipeline (MENAP; http://ieg2.ou.edu/MENA/, Zhou et al., 2010; Deng et al., 2012). Briefly, the fMEN was constructed based on the Pearson correlation matrix calculated by the correlation coefficient (*r* value) between each of the two detected genes (Deng et al., 2012). A series of thresholds was then applied to the matrix and the similarity threshold (st) was kept or matrix eigenvalues were calculated using an RMT-based approach (Deng et al., 2016). During these calculations, the most suitable st was selected to obtain the Poisson distribution of the calculated eigenvalues, which indicates the non-random properties of a complex system (Deng et al., 2016). Each network was then separated into modules by fast greedy modularity optimization. For each network, 100 corresponding random networks were then generated with the same network size and an average number of links. Differences in the indices between the constructed and random networks were determined by statistical *Z* test.

Cytoscape 3.6.1 software (Shannon et al., 2003) and Gephi 0.9.2-beta (Bastian et al., 2009) were applied to visualize the network of the nodes.

### Identification of Node Roles

Topological roles of each functional gene were assessed by the within-module connectivity (Zi) and among-module connectivity (Pi; Guimerà and Amaral, 2005). Nodes in a network were organized into the following four categories: network hubs (Zi > 2.5 and Pi > 0.62), connectors (Pi > 0.62), module hubs (Zi > 2.5), and peripherals (Zi < 2.5 and Pi < 0.62); the former two have important roles in network topology (Zhou et al., 2010).

### Statistical Analyses

Using the microarray analysis pipeline (see text footnote 1), Student’s *t* tests were performed to determine the differences in functional gene diversity and relative signal intensity of each functional gene category and certain subcategories or phylogenetic groups between the HELs and LELs. Three non-parametric multivariate statistical tests, including a permutational multivariate analysis of variance using distance matrices (ADONIS, “Adonis” function), an analysis of similarity (ANOSIM, “anosim” function), and a multiple response permutation procedure (MRPP, “mrpp” function), and detrended correspondence analysis (DCA) were performed to determine the differences in MFS. These four analyses were conducted using the vegan package (version 2.3-0; Oksanen et al., 2013) in the R statistical computing environment.

Canonical correspondence analysis (CCA) was performed to detect the relationships between MFS and environmental parameters because the length of the first DCA axis run on the species data was >2. Forward selection was carried out to determine the significant factors in the CCA model by 999 simulated permutations (*p* < 0.05). All environmental variables were log (*x*+1)-transformed except for the values of water temperature and pH. In addition, variation partitioning analysis (VPA) was performed to identify individual and interactive contributions of productivity (Chl *a* and DOC), space (elevation and lake area), and the rest of the tested environmental heterogeneity to MFS (Li et al., 2017). CCA and VPA were performed using the vegan package in R.

Correlation coefficients between modules and environmental factors were tested on MENAP. The module eigengene E (the first principal component of modules; de Menezes et al., 2015) of the top modules for both the HELs and LELs networks were calculated, and their relationships with environmental variables were then estimated using Spearman’s rank correlation test.

## Results

### Microbial Functional Gene Richness

**FIGURE 1:**
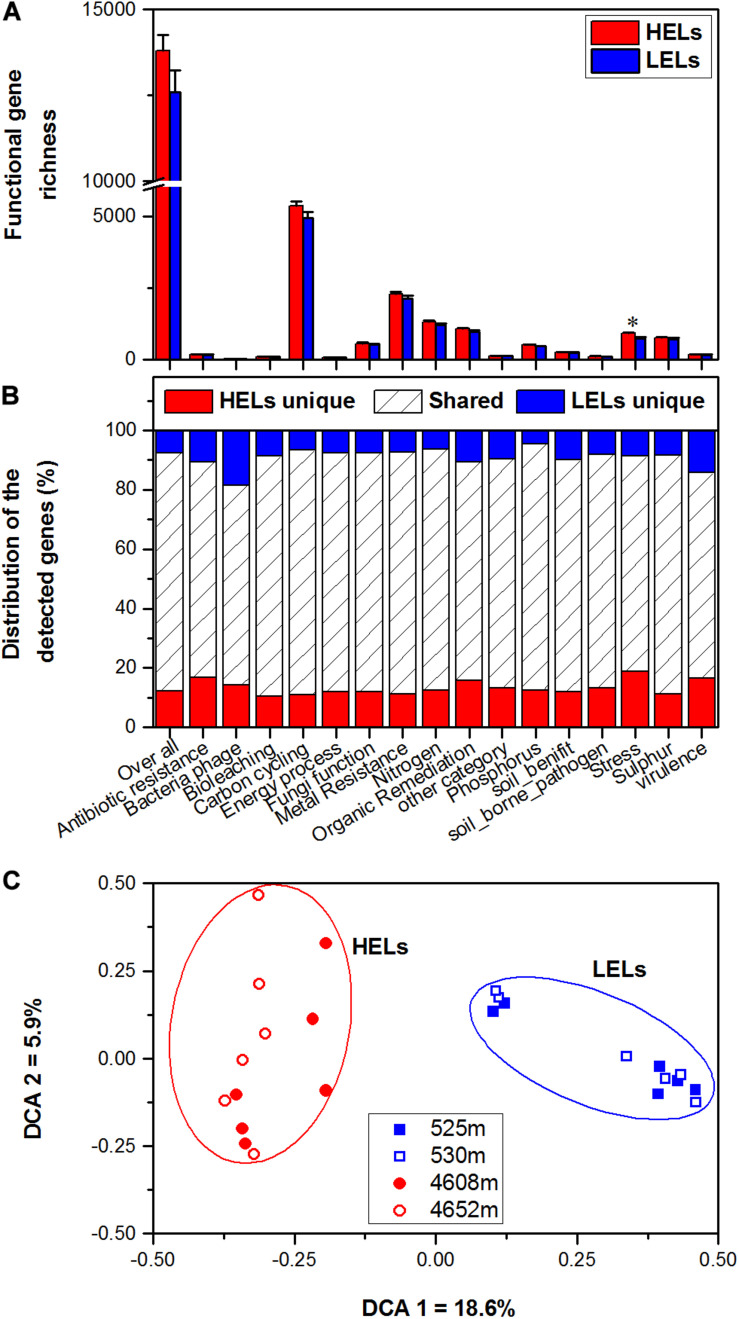
Microbial functional gene richness **(A)**, distribution (shared or unique) of the detected genes **(B)**, and DCA **(C)** of the microbial functional community structure in the two HELs and two LELs involved in certain biogeochemical cycling processes. All data in panels a and b are presented as the mean ± s.e. calculated from 12 biological replicates. Asterisks (*) above the bars indicate significant (*p* < 0.05) differences. The values on axes 1 and 2 in panel **(C)** are percentages of total variations that can be attributed to the corresponding axis.

In total, 17,238 functional genes were detected in the 24 samples, of which 14,912 genes were derived from bacteria, 631 genes were from archaea, 1603 genes were from fungi, and the remaining genes were from bacteriophages. There were no significant differences in overall gene richness between the studied lakes at low and high elevations (*p* > 0.05, Figure 1A). Within all of the detected gene categories (carbon, nitrogen, phosphorus, and sulfur cycling; organic remediation; metal homeostasis; secondary metabolism; stress response; and virulence), only the richness of stress response genes was significantly higher in the HELs than in the LELs (*p <* 0.05 in all cases, Figure 1A), and no significant differences in richness were observed for the other detected gene categories (*p* > 0.05, Figure 1A).

Microbial functional gene overlap between elevations was calculated and revealed that 75.58–87.32% of the genes were shared between the HELs and LELs (Figure 1B and Supplementary Table 2). In the LELs, 7% of the genes were unique, and most of the total genes (81%) and the genes detected in each gene category (73–82%) could be found in the HELs (Figure 1B and Supplementary Table 2), while the HELs had 12% unique genes, most of which were associated with stress resistance (Figure 1B).

### Microbial Functional Gene Structure

The MFS differed markedly between the LELs and HELs. DCA of GeoChip data indicated that the MFSs in the two LELs and those in the two HELs were separated by elevation along DCA axis 1 (Figure 1C). Consistently, three different non-parametric multivariate statistical tests also demonstrated that MFS differed significantly between the HELs and LELs (ADONIS, ANOSIM, and MRPP; *p <* 0.01 in all cases, Table 1) but not within the two HELs or the two LELs (*p* > 0.21 in all cases, Table 1).

**TABLE 1:** Significance tests of the microbial functional gene structure in lakes at low and high elevations.

		MRPP		ANOSIM		Adonis	
		δ	*p*	*R*	*p*	*F*	*p*
525 m vs 4,652 m	Euclidean	56.03	**0.002**	0.64	**0.002**	6.51	**0.001**
	Horn	0.12	**0.003**	0.53	**0.002**	7.56	**0.002**
	Bray	0.14	**0.003**	0.58	**0.001**	7.13	**0.002**
525 m vs 4,608 m	Euclidean	56.26	**0.003**	0.56	**0.002**	0.30	**0.004**
	Horn	0.13	**0.002**	0.45	**0.004**	0.31	**0.005**
	Bray	0.14	**0.006**	0.44	**0.004**	0.30	**0.002**
530 m vs 4,652 m	Euclidean	55.8	**0.002**	0.71	**0.001**	0.39	**0.001**
	Horn	0.12	**0.004**	0.59	**0.005**	0.42	**0.001**
	Bray	0.14	**0.003**	0.58	**0.004**	0.40	**0.002**
530 m vs 4,608 m	Euclidean	56.49	**0.005**	0.58	**0.003**	0.35	**0.003**
	Horn	0.13	**0.006**	0.48	**0.002**	0.38	**0.001**
	Bray	0.14	**0.003**	0.48	**0.001**	0.37	**0.002**
Low vs high	Euclidean	56.32	**0.001**	0.59	**0.001**	11.85	**0.001**
	Horn	0.13	**0.001**	0.48	**0.001**	11.76	**0.001**
	Bray	0.14	**0.001**	0.48	**0.001**	11.13	**0.001**
525 m vs 530 m	Euclidean	57.98	0.304	0.03	0.293	0.04	0.773
	Horn	0.14	0.304	0.02	0.290	0.03	0.755
	Bray	0.15	0.281	0.03	0.279	0.03	0.750
4,608 m vs 4,652 m	Euclidean	54.3	0.233	0.06	0.219	0.05	0.778
	Horn	0.11	0.219	0.04	0.279	0.04	0.739
	Bray	0.13	0.238	0.02	0.317	0.04	0.753

*Three different permutation tests were performed, including multiple response permutation procedure (MRPP), analysis of similarity (ANOSIM), and permutational multivariate analysis of variance (Adonis), based on Euclidean, Horn, or Bray distance. Bold values indicate test results with *p* < 0.05.*

### Functional Genes Involved in Stress Response

**FIGURE 2:**
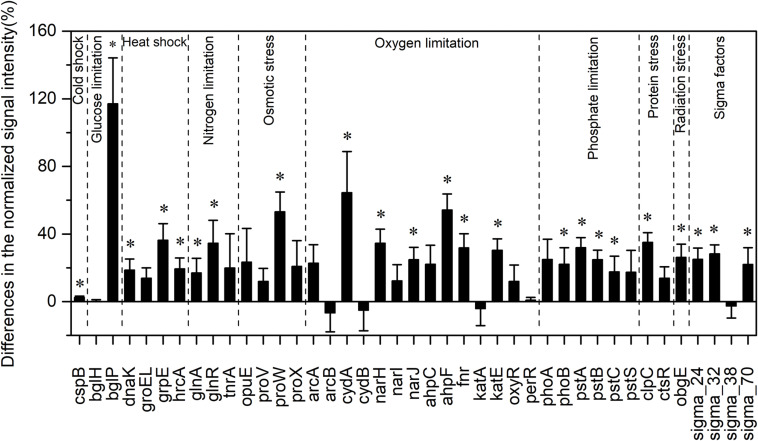
Differences in the normalized intensities of the stress response gene categories between the two LELs and two HELs. All data are presented as mean ± s.e. calculated from biological 12 replicates. Asterisks (*) above the bars indicated significant (*p* < 0.05) differences.

Among all evaluated functional pathways, stress response genes were the most variable, and most of these genes (30 out of 41) had significantly higher normalized signal intensities in the HELs than in the LELs (*p <* 0.05, Figure 2). These functional genes with higher normalized signal intensities in the HELs represented all of the detected stress gene categories including pathway-specific genes for responses to nutrient and oxygen stress, radiation, cold shock, heat shock, and sigma factors. This suggested that the stress response potential in the HELs was greater than that in the LELs. For example, the normalized signal intensities of the functional genes related to glucose limitation (*bglP*), oxygen limitation (*cydA* and *ahpF*), and osmotic stress (*proW*) increased by more than 50% in the HELs compared with those in the LELs (*p <* 0.05 in all cases, Figure 2). In addition, normalized signal intensities of the functional genes related to radiation stress, heat shock, nitrogen limitation, phosphate limitation, sigma factors, and protein stress, such as *obgE*, *grpE*, *glnR*, *pstA*, sigma_32, and *clpC*, increased by more than 20% in the HELs compared with those in the LELs (*p <* 0.05 in all cases, Figure 2).

Taxa–function relationships revealed that most of the differences in the glucose limitation between the HELs and LELs were exclusively derived from *Firmicutes*, which was not found in the LELs (*p <* 0.05, Supplementary Figure 2A). Differences in oxygen limitation between the HELs and LELs were mostly related to *Firmicutes*, *Betaproteobacteria*, *Deltaproteobacteria*, *Gammaproteobacteria*, and fungi (Supplementary Figure 2B). Variations in the pathways related to osmotic stress and nitrogen limitation were largely attributed to a greater abundance of *Gammaproteobacteria* in the HELs (Supplementary Figures 2C, 1E). Differences in heat shock were mostly related to *Actinobacteria* and *Firmicutes* (Supplementary Figure 2D, *p <* 0.05 in both cases). Similarly, cold shock genes derived from *Actinobacteria* had higher normalized signal intensities in the HELs than those in the LELs (Supplementary Figure 2A, *p <* 0.05). Genes indicative of pathways that respond to phosphate limitation were relatively widespread, but particularly abundant among the *Gammaproteobacteria*, *Cyanobacteria*, and *Betaproteobacteria* (Supplementary Figure 2F). Differences in sigma factors were mostly attributed to *Actinobacteria*, *Alphaproteobacteria*, *Cyanobacteria*, *Deinococcus-Thermus* (primarily *Deinococcus deserti* and *Deinococcus geothermalis*), and *Gammaproteobacteria* (Supplementary Figure 2G). Differences in protein stress were mostly attributed to *Firmicutes* (primarily *Exiguobacterium* sp., some strains of which can grow within a wide range of pH and tolerate high levels of UV radiation) and *Verrucomicrobia*, which were only detected in HELs (Supplementary Figure 2H). Differences in radiation stress were related to the most diverse range of taxa, including *Chlorobi*, *Cyanobacteria*, *Deltaproteobacteria*, *Deinococcus-Thermus*, *Firmicutes*, *Gammaproteobacteria*, and *Thermotogae* (Supplementary Figure 2I).

### Functional Genes Involved in Carbon Cycling

**FIGURE 3:**
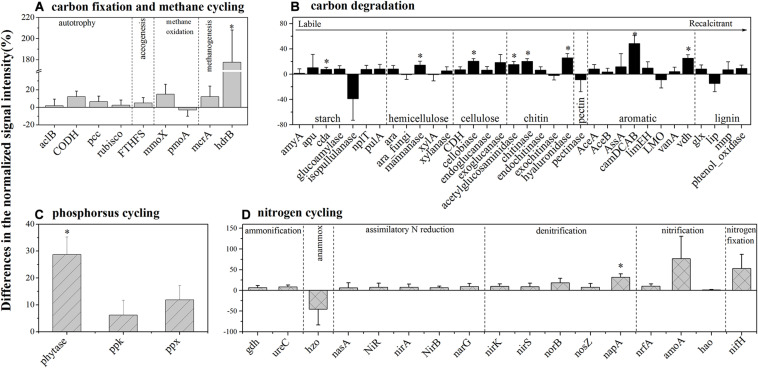
Differences in the normalized intensities of certain biogeochemical cycling processes between the two LELs and two HELs. **(A)** Each subcategory of the carbon fixation and methane cycling; **(B)** each subcategory of carbon degradation; **(C)** each subcategory of phosphorus cycling; and **(D)** each subcategory of nitrogen cycling. All data are presented as mean ± s.e. calculated from biological 12 replicates. Asterisks (*) above the bars indicated significant (*p* < 0.05) differences.

For autotrophic carbon fixation genes, although ATP citrate lyase (*aclB*), carbon-monoxide dehydrogenase (*CODH*), ribulose-1,5-bisphosphate carboxylase/oxygenase (*rubisco*), and acetyl-CoA carboxylase biotin carboxylase subunit (*pcc*) gene groups were observed in both HELs and LELs, the normalized signal intensities of all of them were not significantly different (Figure 3A, *p* > 0.20 in all cases). Similarly, the metabolic potential of acetogenesis [i.e., formyltetrahydrofolate synthetase (*FTHFS*)] in the Wood–Ljungdahl pathway was also not significantly changed with the increase of elevation (Figure 3A, *p* = 0.43). The same pattern was observed in methane oxidation genes of *mmoX* and *pmoA* (Figure 3A, *p* > 0.22 in both cases). However, the normalized signal intensity of a methanogenesis gene, *hdrB* (methyl-coenzyme M reductase), was nearly twofold higher in the HELs compared with the LELs (Figure 3A, *p <* 0.05).

Taxa–function relationships revealed that most of these differences in carbon fixation genes were derived from *Euryarchaeota* and *Crenarchaeota* and that these *Euryarchaeota* (primarily *Archaeoglobus fulgidus* and *Methanolinea tarda*) and *Crenarchaeota* (*Metallosphaera yellowstonensis*) were not detected in the LELs (Supplementary Figure 3A).

In contrast to the carbon-fixing genes, eight genes involved in carbon degradation, especially the metabolic potentials of recalcitrant carbon such as chitin, and aromatic compounds, had significantly different normalized signal intensities between the HELs and the LELs (Figure 3B). Compared with the LELs, the most significantly enhanced carbon degradation genes in the HELs were related to aromatic metabolism (*camDCAB*, increased 48%), followed by another aromatic metabolizing gene [vanillin dehydrogenase (*vdh*), increased 25%], three genes involved in chitin metabolism (*hyaluronidase*, *acetylglucosaminidase*, and *chitinase*, increased 26, 20, and 15%, respectively), and one involved in cellulose metabolism (*cellobiase*, increased 20%). Other markedly increased normalized signal intensities in the HELs were detected in a starch degradation gene [cyclomaltodextrinase (*cda*), increased 8%] and a hemicellulose degradation gene (*mannanase*, increased 14%; *p <* 0.05 in all cases). These differences were ascribed to some common carbon degradation microorganisms, including *Firmicutes*, *Actinobacteria*, *Gammaproteobacteria*, *Alphaproteobacteria*, and *Betaproteobacteria* (Supplementary Figures 3B–I).

### Functional Genes Involved in Phosphorus and Nitrogen Metabolism

Among the three phosphorus cycling genes, only the functional potential of *phytase* for phytate degradation was significantly higher in HELs than in LELs (*p <* 0.05, Figure 3C). This was in accordance with the higher relative abundance of polyphosphate-degrading microorganisms from *Gammaproteobacteria*, *Firmicutes*, *Alphaproteobacteria*, *Betaproteobacteria*, and *Bacteroidetes* in the HELs (*p <* 0.05, Supplementary Figure 3K). Regarding the 17 nitrogen cycling gene groups, only one nitrate reduction gene (*napA*) had a significantly higher normalized signal intensity in the HELs compared with those in the LELs (*p <* 0.05, Figure 3D). Taxa–function relationship analysis revealed that this marked difference in normalized signal intensity for nitrate reduction between HELs and LELs was observed primarily in *Actinobacteria* and *Deltaproteobacteria* (*p <* 0.05, Supplementary Figure 3J).

### Microbial Functional Gene Network Analysis

**FIGURE 4:**
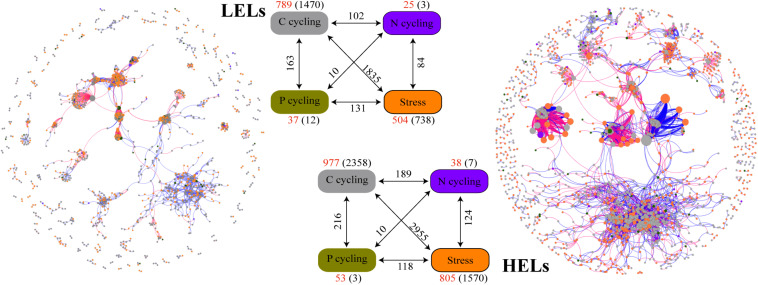
Differences in network interactions of these genes between the two LELs and two HELs. Network analysis showing the associations within each gene category and associations between different gene categories. A connection indicates a strong (Spearman’s *r* > 0.8 or *r* < -0.8) and significant (*p* < 0.01) correlation. A blue line indicates a positive interaction between two nodes, and a red line indicates a negative interaction. The size of each node is proportional to the number of connections (i.e., degree). Colors of the nodes indicate different functional gene categories: orange, stress response; gray, carbon cycling; dark violet, nitrogen cycling; dark goldenrod, and phosphorus cycling. Numbers outside and inside parentheses represent the node numbers and inner connections belonging to the corresponding gene category (i.e., there were 504 nodes and 738 inner connections for stress response genes in LELs); the numbers adjacent to edge connections represent cross-gene-category interactions.

The two networks generated in this study produced 1873 nodes and 7550 links (47% negative and 53% positive) for the HELs and 1355 nodes and 4548 links (45% negative and 55% positive) for the LELs, respectively (Figure 4). Typical correlation values of the two constructed fMENs were both 0.98 (Table 2), indicating that connectivity of the two fMENs fitted the power law model. The indexes including modularity, clustering coefficients, and harmonic geodesic distance were significantly different from those of the corresponding random networks for the lakes at both elevations (Table 2). This indicated that the fMENs in both lakes were non-random (*p <* 0.001 in all cases, Table 2). Compared with the network of the LELs, the fMEN in the HELs generally had higher connectivity, shorter geodesic distances, higher clustering efficiencies, and more modules (Table 2).

**TABLE 2:** Major topological properties of the empirical fMENs and their associated random networks in the LELs and HELs.

Elevation of Lakes (m)	No. of original genes^a^	Empirical networks									Random networks^c^		
		Similarity threshold (St)	Network size (*n*)^b^	Links	*R*^2^ of power law	Average connectivity (avgK)	Harmonic geodesic distance (HD)	Average clustering coefficient (avgCC)	Modularity (no. of modules)	Transitivity (Trans)	Harmonic geodesic distance (HD ± SD)	Average clustering coefficient (avgCC ± SD)	Average modularity (M ± SD)
LELs	1,808	0.98	1,355	4,548	0.881	6.713	6.668^d^	0.350^d^	0.790^d^(133)	0.333	3.408 ± 0.014	0.029 ± 0.003	0.333 ± 0.003
HELs	2,397	0.98	1,873	7,550	0.877	8.062	6.269^d^	0.366^d^	0.865^d^(156)	0.340	3.379 ± 0.009	0.023 ± 0.002	0.340 ± 0.002

*^*a*^ The number of genes used for network construction. ^*b*^ The number of nodes in a network in a functional molecular ecological network. ^*c*^ The parameters were generated from 100 times of random rewired networks. ^*d*^ Significant difference between the empirical network and the random network (*p* < 0.001, *Z* test).*

More interactions and module hubs in relation to stress response and carbon cycling functional genes were found in the network of the HELs than in that of the LELs (Supplementary Table 3). Hub nodes in the LELs network predominantly belonged to carbon cycling genes (i.e., carbon degradation subcategory; Supplementary Table 3). In contrast, most hub nodes in the HELs network belonged to stress response genes (i.e., oxygen stress, oxygen limitation, sigma factors, and phosphate limitation), and some belonged to carbon cycling genes (Supplementary Table 3). No connectors were detected in the LELs network, but two were present in the HELs network, one of which was a stress response gene (Supplementary Table 3). The top-ranked functional genes, whose connectivity (links between genes) ranked in the top five of each network, in the HELs network were distinct from those in the LELs (Supplementary Figure 4). The top five genes in the HELs network belonged to stress response genes (*katE*, *fnr*, and *sigma_24*) and carbon cycling genes (*cellobiose* and *hyaluronidase*), while those in the network from the LELs belonged to carbon cycling genes (*chitinase* and *hyaluronidase*), a nitrogen reduction gene (*napA*), and a stress response gene (*sigma_70*). Moreover, the interactions of the top five genes in the HELs network (Supplementary Figure 4A) were much more complex in terms of network size and connectivity than those in the LELs network (Supplementary Figure 4B).

### Linkages Between MFS and Lake Environmental Parameters

**FIGURE 5:**
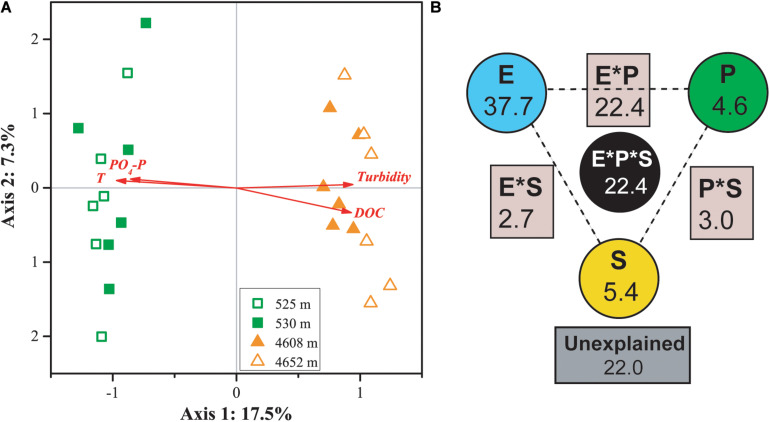
CCA **(A)** and VPA **(B)** of relationships between the microbial functional community structure and the environmental variables. P: productivity: Chl *a*, DOC; S: space: elevation and area; and E: the rest of the tested environmental variables. Numbers on the CCA axis and in the VPA diagram show the percentages of explained variations in the microbial functional gene profile. In the VPA diagram, the edges of the triangle presented the variation explained by each factor alone, while the sides of the triangles presented interactions of any two factors and the middle of the triangles represented interactions of all three factors.

Canonical correspondence analysis indicated that four factors—temperature, PO_4_-P, DOC, and turbidity—played key roles in shaping the variance of the differences in MFS in the four lakes (for all canonical axes, *p <* 0.05, Figure 5A). The first two axes explained 19.8% of the observed variation in the composition of the MFS, and 37.6% was explained by all four canonical axes. All four selected factors showed high canonical correlations with the first axis.

Variation partitioning analysis indicated that environmental heterogeneity was most important in explaining the variance of the differences in MFS in the four lakes (Figure 5B). The three categories of variables—environmental heterogeneity, productivity, and space (elevation and lake area)—explained 37.7, 4.6, and 5.4% of the total variance, respectively. Interactions among the three groups contributed 30.3% of the total variance. A smaller portion (22%) of the MFS variations could not be explained by the tested variables.

The five top-ranking modules detected in the HELs and LELs networks demonstrated different relationships with the environmental variables (Supplementary Figure 5). In the network of the HELs, modules 1 and 5 were positively correlated with turbidity; modules 3 and 4 were negatively correlated with water temperature, but the concentrations of Chl *a* and DOC were positively correlated with pH; and module 2 was negatively correlated with oxidation reduction potential. In contrast, in the network of the LELs, the largest module, module 1, showed positive and negative correlations with water temperature and total phosphorus, respectively. Modules 3 and 4, which were negatively correlated with DOC, showed positive correlations with DO, while module 2 did not correlate with any of the investigated environmental variables.

## Discussion

### Enhanced Potentials of Stress Response in the HELs

In this study, two LELs were used as a reference to investigate the responses of microbial functional traits to elevational increase. Microbial metabolic potentials were significantly different between the HELs and the LELs. The biggest difference was that the normalized signal intensities of microbial stress genes were higher in the HELs than in the LELs (Figure 1). Soil microbial communities at higher elevations were previously shown to maintain high normalized signal intensities in stress response genes including those involved in cold shock, oxygen limitation, and osmotic stress (Yang et al., 2014). In addition to these genes, another seven subcategories of stress response genes with higher normalized signal intensities in the HELs were identified in the present study; these were nitrogen limitation, phosphate limitation, glucose limitation, radiation stress, heat shock, protein stress, and sigma factors (Figure 2). Previous studies using different versions of GeoChip to this study detected all the probes related to these seven subcategories of stress response genes (Tu et al., 2014; Picazo et al., 2020); these studies used GeoChip 4.0, the same as that used by Yang et al. (2014). Detection of more stress response genes with higher normalized signal intensities in the HELs in the present study may be due to more significant changes in environmental factors of the water within a broader elevation gradient and to the characteristics of the lakes themselves. With the increase in elevation from approximately 530 m to more than 4,600 m, the water temperature dropped significantly from 27 to 3°C; Chl *a* concentration decreased more than 20-fold; the concentrations of nutrients such as phosphate, nitrate, and ammonium were negligible; and DO decreased by 50% (Supplementary Table 1). These environmental conditions in the HELs could cause higher microbial metabolic potentials of nitrogen limitation, phosphate limitation, glucose limitation, and protein stress genes.

The normalized signal intensities of microbial radiation stress genes were also significantly higher in the HELs compared with those in the LELs. This was in contrast to data from grass soil in which radiation stress genes were more abundant at the lower elevation (Yang et al., 2014). This difference might be attributed to the fact that UV radiation is more intensive in surface water than in grass soil where aboveground vegetation prevents UV penetration (Yang et al., 2014). The discovery of more functional genes with high normalized signal intensities in diverse subcategories of the stress response pathways in the HELs than in grass soil confirms that recruitment of microbes to exposed habitats is strongly based on selection of stress tolerance traits. Furthermore, this finding indicates that these microbial communities have more diverse abilities with which to tolerate harsh environmental conditions than more sheltered habitats (Chan et al., 2013).

Higher normalized signal intensities in the microbial functional genes related to heat shock and sigma factors were observed in the HELs compared with the LELs. Similar results were also found on Antarctica rock surface (Chan et al., 2013), in Tibetan mountainous grass soil (Yang et al., 2014; Qi et al., 2017), and on stones in streams (Picazo et al., 2020). Mounting evidence suggests that besides protecting microbial cells from environmental insults of sudden temperature increases, the transcriptional activities of heat shock genes are maintained at a steady-state level that is frequently greater than the initial basal level for the purpose of facilitating microbial growth under non-optimal environmental conditions (Macario et al., 1999; Roncarati and Scarlato, 2017). Moreover, some heat shock proteins are also required and are abundant during normal growth conditions. For example, the genes *GroEL* and *dnaK* are instrumental in protein folding even during non-stressed growth conditions, although their activities become more important during stress (Macario et al., 1999; Roncarati and Scarlato, 2017). In addition to temperature variations, several other stress conditions, such as osmotic changes, desiccation, antibiotics, solvents, and heavy metals, can elicit heat shock responses (Roncarati and Scarlato, 2017). In the present study, HELs are frozen for most of the year. However, there are diurnal temperature variations in the summer, which are likely to cause an increase in heat shock genes. Alternative sigma factors are the positive transcriptional regulators of heat shock genes (Roncarati and Scarlato, 2017), and these were also increased in the HELs in the present study.

### Enhanced Potentials of Carbon Degradation in the HELs

There were no significant changes in the normalized signal intensities of the functional gene categories of carbon, phosphorus, and nitrogen metabolisms in the HELs (Figure 1A). However, there were a few marked increases in the normalized signal intensities of carbon degradation genes at the single functional gene level (Figure 3B). Differences in the composition of DOC may be a principal factor causing changes in microbial taxonomic structure in aquatic ecosystems (Teeling et al., 2012; Sarmento et al., 2013), and this may subsequently influence the microbial functional metabolic potentials (Kuang et al., 2016). Generally, there are two main carbon sources in lake ecosystems: terrestrial (allochthonous) inputs of carbon received from the landscape around lakes and autochthonous carbon provided by in-lake primary production (Rose et al., 2015). The relative contribution of allochthonous carbon inputs increases with increasing elevation whereas their lability decreases with increasing elevation (Zhou et al., 2018). Zhou et al. (2019) reported that dissolved organic matter aromaticity and the relative abundance of lignin compounds in glacial-fed streams and rivers on the Tibetan Plateau all increased during water flows downstream from the glacial terminus, and the same may apply in the HELs in the present study. The DOC-to-Chl *a* ratio is an allochthony indicator (Carpenter et al., 2005; Bade et al., 2007; Pace et al., 2007). In the present study, the DOC-t- Chl *a* ratios in the two HELs were on average 12-fold greater than those in the two LELs (Supplementary Table 1). The more allochthonous DOC in the HELs may promote the microbial functional potentials of carbon degradation, such as *camDCA*, *chitinaseb*, *hyaluronidase*, *cellobiase*, *cda*, and *mannanase* (Figure 3B).

### Enhanced Interactions Among the Co-occurring Functional Genes in the HELs

Comparison of network properties, such as links, network size, connectivity, and clustering coefficients, in the lakes at the two elevations identified reinforced complexity in the HELs network. Soil microbial networks detected in northern China were previously reported to be more complex than those found in southern China, suggesting that temperature affects the complexity of microbial networks at a continental scale (Ma et al., 2016; Zhang et al., 2018). Network analyses in the current study indicated that, with the increase in elevation from approximately 530 to 4,600 m, the functional gene network increased in network size and became much more connected and clustered (Table 2 and Figure 4). Temperature and DOC were two of the most important environmental causes of these changes in network properties (Supplementary Figure 5). This finding was consistent with that of acidic mining lakes, in which harsh conditions such as low pH, high concentrations of sulfate and metals, and limited carbon, nitrogen, and phosphorus sources resulted in more complex microbial functional gene networks (Ren et al., 2018). These discoveries may be attributed to the fact that to survive in harsh environments, microorganisms cooperate by participating in diverse metabolic pathways of the microbial food chain, such as degrading (complex) organics or metabolizing carbon, nitrogen, and phosphorus (Dong et al., 2020). Similarly, to adapt to the high elevation and its related factors, some microorganisms in the HELs enhanced their potentials of recalcitrant carbon degradation genes (such as *cellobiase_bact_arch*, *chitinaseb*, and *hyaluronidase*; Figure 3B) to provide carbon sources for other microorganisms. In return, other microbes increased their potentials of stress response genes (e.g., *katE*, *fnr*, and sigma_24 and sigma_70 genes; Figure 2) to help other microorganisms survive under conditions of low oxygen, low temperature and large variation of temperature, and high UV radiation. These results were confirmed by the HELs network having more interactions and also containing more module hubs and two connectors in relation to stress response and carbon cycling functional genes, compared with the LELs network (Supplementary Table 3 and Supplementary Figure 4). A previous report confirmed that one member of nodes that had high connections in the bacterial co-occurrence network could facilitate one member of the peripheral vertexes by providing the latter with several low-molecular-weight organic substrates (Xian et al., 2020). However, the mechanisms of our findings in the present study require further investigation using a combination of culture-dependent and culture-independent techniques, such as metatranscriptomics, metaproteomics, and metabolomics approaches.

### No Differences in Overall Functional Gene Richness Between the HELs and LELs

The overall functional gene diversity was not significantly different between the HELs and LELs. This is in contrast to data from grass soil where the number of detected microbial genes at the 3,200-m site was approximately half of those at the three higher elevations (3,400, 3,600, and 3,800 m; Yang et al., 2014). These differences may predominantly be due to covariance of elevation and regional characteristics in terrestrial ecosystems, whereas lakes are more homogeneous (Li et al., 2017). For example, the pH in soil changed more within the elevational range of 600 m (Yang et al., 2014) than in lake water within the elevational range of 4,100 m (this study), and pH is known to have an adverse effect on microbial gene diversity (Yang et al., 2014; Ren et al., 2018). Furthermore, large numbers of introduced rare bacterial taxa, which enter lakes from surrounding catchments or sediments, can increase the overall bacterial diversity in small lakes with large catchment areas at high elevations (e.g., Lindström and Bergström, 2004; Crump et al., 2012; Li et al., 2017) and are also likely to influence microbial functional gene diversity. Thus, in comparison to the LELs, the microbial gene richness in the two HELs did not decrease but increased slightly.

## Conclusion

This study demonstrated that the elevation increase of approximately 4,100 m, which relates to changes in different environmental variables, has a strong influence on the profile of microbial functional potentials in lake water columns. Harsh conditions (e.g., nutrient limitation, low temperature and large variation of temperature, high UV radiation, and more recalcitrant DOC) at high elevations may significantly promote the microbial metabolic potentials among functional genes involved in stress responses and recalcitrant carbon degradation in freshwater lakes. Microbes exposed to higher elevation stressors had increased signal intensities of stress response functional genes, increased numbers of module hubs of these genes, and enhanced complexity of gene interactions. This study highlights that the limnetic microbial communities develop functional strategies to cope with the harsh conditions at high elevations.

## Data Availability Statement

The datasets presented in this study can be found in online repositories. The names of the repository/repositories and accession number(s) can be found below: www.ncbi.nlm.nih.gov/geo/query/acc.cgi?acc=GSE156784.

## Author Contributions

HL, JZ, and QW conceived and initiated the study and led the writing of the manuscript. LR collected the samples. QY analyzed the data. All the authors contributed substantially to the study.

## Conflict of Interest

The authors declare that the research was conducted in the absence of any commercial or financial relationships that could be construed as a potential conflict of interest. The reviewer KX declared a shared affiliation with one of the authors QW to the handling editor at the time of review.

## Footnotes

1 http://ieg.ou.edu/microarray/
