# Supplementary material for: Enhanced Metabolic Potentials and Functional Gene Interactions of Microbial Stress Responses to a 4,100-m Elevational Increase in Freshwater Lakes
Source: Front Microbiol. 2021 Jan 13;11:595967. doi: 10.3389/fmicb.2020.595967 (PMC7838385; doi:10.3389/fmicb.2020.595967)
Supplement: Supplementary file 1 [file Table_1.DOC]

**Online Supporting Materials For**

**Enhanced metabolic potentials and functional gene interactions of microbial stress response to a 4,100-m elevational increase in freshwater lakes**

Huabing Li ^1^, Jin Zeng ^1, *^, Lijuan Ren ^2^, Qingyun Yan^3^, Qinglong L. Wu^1, 4, *^

^1^ State Key Laboratory of Lake Science and Environment, Nanjing Institute of Geography and Limnology, Chinese Academy of Sciences, East Beijing Road 73, Nanjing 210008, People's Republic of China

^2^ Department of Ecology and Institute of Hydrobiology, Jinan University, Guangzhou 510632, China

^3^ Environmental Microbiomics Research Center, School of Environmental Science and Engineering, Southern Marine Science and Engineering Guangdong Laboratory (Zhuhai), Sun Yat-sen University, Guangzhou 510006, China

^4^ Sino-Danish Centre for Education and Research, University of Chinese Academy of Sciences, Beijing, China

^*^ Correspondence author: Qinglong L. Wu, Email: [qlwu@niglas.ac.cn](mailto:qlwu@niglas.ac.cn), or [jzeng@niglas.ac.cn](mailto:jzeng@niglas.ac.cn) State Key Laboratory of Lake Science and Environment, Nanjing Institute of Geography and Limnology, Chinese Academy of Sciences, East Beijing Road 73, Nanjing 210008, People's Republic of China.

Running title: Microbial functional response to increasing-elevation

**Table S1.** Summary of location and environmental characteristics of the investigated lakes at low and high elevations at Siguniang Mountain in China^a^

|  | Lake name (Abbreviation) | | | |
| --- | --- | --- | --- | --- |
| Variables | Maojakou (MJK) | Baigongyan (BGY) | Heihaizi (HHZ) | Baihaizi (BHZ) |
| Group | Low elevation lakes (LELs) | | High elevation lake (HELs) | |
| Position (°N, °E) | 30.587,  104.310 | 30.589,  104.311 | 31.093,  102.958 | 31.098,  102.956 |
| Ele (m) | 525 | 530 | 4608 | 4652 |
| Area (ha) | 14.3 | 29.9 | 2.1 | 1.1 |
| Maximum depth (m) | 5.7 | 9.6 | 19 | 6.7 |
| T (°C) | 25.64±0.13 | 27.17±0.34 | 3.7±0.15 | 1.86±0.77 |
| TN (mg/L) | 7.99±0.12 | 4.43±0.11 | 0.2±0.07 | 0.19±0.01 |
| TP (mg/L) | 0.13±0.01 | 0.07±0 | 0.01±0 | 0.01±0 |
| Nitrate (mg/l) | 5±0.09 | 2.51±0.11 | 0.14±0 | 0.12±0.01 |
| Nitrite (mg/l) | 0.06±0.01 | 0.01±0 | 0±0 | 0.02±0.01 |
| PO_4_-P(ug/L) | 34.26±12.89 | 8.1±1.04 | 2.33±0.38 | 1.65±0.09 |
| Ammonium (mg/L) | 0.2±0.05 | 0.24±0.06 | 0.01±0 | 0±0 |
| Chl *a* (ug/L) | 108.9±12.62 | 26.35±2.35 | 3.2±0.32 | 3±0.13 |
| DOC (mg/L) | 21.05±1.37 | 25.26±1.56 | 12.96±0.5 | 9.72±1.7 |
| Turbidity (NTU) | 8.28±0.67 | 22.07±0.77 | 1.23±0.06 | 1.07±0.23 |
| Salinity (ppt) | 0.24±0 | 0.17±0 | 0.03±0 | 0.04±0 |
| COND (mS/cm) | 0.52±0 | 0.38±0 | 0.04±0 | 0.04±0 |
| DO (mg/L) | 14.36±1.6 | 15.6±0.2 | 6.69±0.13 | 7.02±0.04 |
| pH | 8.79±0.14 | 9.18±0.01 | 8.12±0.02 | 8.04±0.03 |
| ORP (mV) | 388.6±6.45 | 373.82±0.9 | 603.28±18.47 | 609.73±12.52 |

^a^ Ele, elevation; T, water temperature; TN, total nitrogen; TP, total phosphorus; PO_4_-P, soluble phosphorus; Chl *a*, chlorophyll a; DOC, dissolved organic carbon; COND, conductivity; DO, dissolved oxygen; ORP, oxidation reduction potential. The results are represented as the mean ± standard error.

**Table S2**. Percentages of overlapping microbial functional genes in the lakes at low and high elevations. The abbreviation of each lake was shown in Table S4

|  | MJK1 | MJK2 | MJK3 | MJK4 | MJK5 | MJK6 | BGY1 | BGY2 | BGY3 | BGY4 | BGY5 | BGY6 | HHZ1 | HHZ2 | HHZ3 | HHZ4 | HHZ5 | HHZ6 | BHZ1 | BHZ2 | BHZ3 | BHZ4 | BHZ5 |
| --- | --- | --- | --- | --- | --- | --- | --- | --- | --- | --- | --- | --- | --- | --- | --- | --- | --- | --- | --- | --- | --- | --- | --- |
| MJK2 | 84.37 |  |  |  |  |  |  |  |  |  |  |  |  |  |  |  |  |  |  |  |  |  |  |
| MJK3 | 81.00 | 93.96 |  |  |  |  |  |  |  |  |  |  |  |  |  |  |  |  |  |  |  |  |  |
| MJK4 | 84.70 | 79.98 | 78.29 |  |  |  |  |  |  |  |  |  |  |  |  |  |  |  |  |  |  |  |  |
| MJK5 | 84.35 | 96.24 | 93.86 | 79.90 |  |  |  |  |  |  |  |  |  |  |  |  |  |  |  |  |  |  |  |
| MJK6 | 83.17 | 86.62 | 86.32 | 81.44 | 86.68 |  |  |  |  |  |  |  |  |  |  |  |  |  |  |  |  |  |  |
| BGY1 | 86.17 | 83.47 | 80.69 | 82.03 | 83.20 | 82.09 |  |  |  |  |  |  |  |  |  |  |  |  |  |  |  |  |  |
| BGY2 | 82.04 | 93.78 | 91.86 | 77.93 | 93.31 | 84.75 | 83.81 |  |  |  |  |  |  |  |  |  |  |  |  |  |  |  |  |
| BGY3 | 80.70 | 91.45 | 91.42 | 78.28 | 91.62 | 84.10 | 80.74 | 91.80 |  |  |  |  |  |  |  |  |  |  |  |  |  |  |  |
| BGY4 | 81.70 | 79.76 | 77.89 | 88.12 | 79.42 | 79.31 | 81.39 | 79.23 | 79.11 |  |  |  |  |  |  |  |  |  |  |  |  |  |  |
| BGY5 | 81.83 | 94.27 | 92.14 | 78.59 | 94.79 | 84.49 | 84.27 | 93.90 | 91.70 | 80.04 |  |  |  |  |  |  |  |  |  |  |  |  |  |
| BGY6 | 82.51 | 90.10 | 89.12 | 79.67 | 90.36 | 84.07 | 81.77 | 89.69 | 90.26 | 80.54 | 90.15 |  |  |  |  |  |  |  |  |  |  |  |  |
| HHZ1 | 87.29 | 81.84 | 78.88 | 83.60 | 81.77 | 80.59 | 83.11 | 80.00 | 79.55 | 80.93 | 80.39 | 80.84 |  |  |  |  |  |  |  |  |  |  |  |
| HHZ2 | 81.16 | 86.20 | 84.02 | 76.46 | 85.43 | 80.69 | 81.37 | 85.93 | 83.03 | 75.87 | 84.70 | 82.49 | 83.27 |  |  |  |  |  |  |  |  |  |  |
| HHZ3 | 80.18 | 87.18 | 85.33 | 76.44 | 86.83 | 80.73 | 79.96 | 86.09 | 84.41 | 76.59 | 86.28 | 83.74 | 83.18 | 93.57 |  |  |  |  |  |  |  |  |  |
| HHZ4 | 84.07 | 84.61 | 82.58 | 83.52 | 84.33 | 81.59 | 83.76 | 83.20 | 81.52 | 83.70 | 83.53 | 82.63 | 86.45 | 86.34 | 87.50 |  |  |  |  |  |  |  |  |
| HHZ5 | 80.72 | 87.17 | 84.44 | 77.02 | 86.89 | 80.29 | 80.93 | 86.01 | 83.42 | 76.98 | 86.57 | 83.21 | 84.52 | 93.18 | 94.94 | 87.71 |  |  |  |  |  |  |  |
| HHZ6 | 83.07 | 86.09 | 84.55 | 81.27 | 85.58 | 83.80 | 81.82 | 84.84 | 83.64 | 80.86 | 84.49 | 83.89 | 85.74 | 88.67 | 89.97 | 88.70 | 89.32 |  |  |  |  |  |  |
| BHZ1 | 84.80 | 79.87 | 76.67 | 81.51 | 79.74 | 77.48 | 85.32 | 78.70 | 76.80 | 81.27 | 79.40 | 77.84 | 86.68 | 82.32 | 81.67 | 86.54 | 83.57 | 83.26 |  |  |  |  |  |
| BHZ2 | 80.52 | 87.32 | 84.75 | 76.82 | 86.96 | 80.54 | 80.79 | 86.46 | 83.90 | 76.74 | 86.27 | 83.65 | 83.71 | 93.23 | 95.03 | 87.75 | 94.81 | 89.34 | 83.79 |  |  |  |  |
| BHZ3 | 79.63 | 84.98 | 84.81 | 75.87 | 84.66 | 80.02 | 80.28 | 85.07 | 83.59 | 75.58 | 84.30 | 82.51 | 80.15 | 91.76 | 92.34 | 85.15 | 90.79 | 86.96 | 80.56 | 92.91 |  |  |  |
| BHZ4 | 83.14 | 84.14 | 81.32 | 80.22 | 83.94 | 79.32 | 83.61 | 83.26 | 81.94 | 80.03 | 83.42 | 82.37 | 86.79 | 87.70 | 88.15 | 88.53 | 88.80 | 86.83 | 88.62 | 89.87 | 86.78 |  |  |
| BHZ5 | 80.76 | 86.52 | 83.62 | 77.70 | 86.54 | 79.59 | 81.44 | 85.56 | 83.45 | 78.07 | 86.51 | 83.36 | 83.96 | 90.83 | 92.63 | 87.28 | 94.22 | 87.81 | 84.60 | 94.40 | 90.12 | 89.43 |  |
| BHZ6 | 81.06 | 85.42 | 82.86 | 81.14 | 84.88 | 81.00 | 82.25 | 83.91 | 82.02 | 81.39 | 84.25 | 82.26 | 84.31 | 87.70 | 89.72 | 89.57 | 89.95 | 89.38 | 86.12 | 91.39 | 87.27 | 88.63 | 90.02 |

**Table S**3 Hubs and connectors of module 1 to module 5 in microbial functional gene networks of low-elevation lakes (LELs) and high-elevation lakes (HELs)

| Objects | Hubs | Node | Gene ID | Module | Degree | Gene name | Gene category | Gene subcategory | Kingdom/Phylum | Genus |
| --- | --- | --- | --- | --- | --- | --- | --- | --- | --- | --- |
| LELs | Module  Hubs | 1 | 334136562 | 1 | 56 | *chitinase_general_bact_arch* | Carbon cycling | Carbon degradation | Firmicutes | Paenibacillus |
|  |  | 2 | 110168086 | 1 | 53 | *pstA* | Stress | Phosphate limitation | Cyanobacteria | erythraeum |
|  |  | 3 | 1771343 | 1 | 53 | *napA* | Nitrogen | Dissimilatory N reduction | Proteobacteria | Moraxella |
|  |  | 4 | 85711354 | 1 | 52 | *phytase* | Phosphorus | Phosphorus utilization | Proteobacteria | Idiomarina |
|  |  | 5 | 108460873 | 1 | 52 | *chitinase_general_bact_arch* | Carbon cycling | Carbon degradation | Proteobacteria | Myxococcus |
|  |  | 6 | 381179197 | 1 | 52 | *mannanase* | Carbon cycling | Carbon degradation | Spirochaetes | Treponema |
|  |  | 7 | 338534120 | 5 | 20 | *chitinase_general_bact_arch* | Carbon cycling | Carbon degradation | Proteobacteria | Myxococcus |
|  |  | 8 | 228848109 | 1 | 18 | *fnr* | Stress | Oxygen stress | Firmicutes | Bacillus |
|  |  | 9 | 189464527 | 3 | 17 | *acetylglucosaminidase_bact_arch* | Carbon cycling | Carbon degradation | Bacteroidetes | Bacteroides |
|  |  | 10 | 269121796 | 4 | 11 | *cellobiase_bact_arch* | Carbon cycling | Carbon degradation | Fusobacteria | Sebaldella |
| HELs | Module  Hubs | 1 | 283455856 | 2 | 116 | *cellobiase_bact_arch* | Carbon cycling | Carbon degradation | Actinobacteria | Bifidobacterium |
|  |  | 2 | 257806866 | 2 | 113 | *hyaluronidase* | Carbon cycling | Carbon degradation | Firmicutes | Enterococcus |
|  |  | 3 | 119474019 | 2 | 101 | *katE* | Stress | Oxygen stress | Fungi | Trichocomaceae |
|  |  | 4 | 197087547 | 3 | 66 | *fnr* | Stress | Oxygen stress | Proteobacteria | Geobacter |
|  |  | 5 | 116611450 | 2 | 64 | *sigma_24* | Stress | Sigma factors | Actinobacteria | Arthrobacter |
|  |  | 6 | 86554932 | 2 | 62 | *pstB* | Stress | Phosphate limitation | Cyanobacteria | Unclassified bacteria |
|  |  | 7 | 160897573 | 3 | 43 | *fnr* | Stress | Oxygen stress | Proteobacteria | Delftia |
|  |  | 8 | 167587540 | 3 | 43 | *katE* | Stress | Oxygen stress | Proteobacteria | Burkholderia |
|  |  | 9 | 85068532 | 3 | 43 | *katE* | Stress | Oxygen stress | Fungi | Sordariaceae |
|  |  | 10 | 145574778 | 3 | 43 | *sigma_24* | Stress | Sigma factors | Proteobacteria | Pseudomonas |
|  |  | 11 | 83949442 | 3 | 43 | *sigma_32* | Stress | Sigma factors | Proteobacteria | Roseovarius |
|  |  | 12 | 83846976 | 3 | 43 | *sigma_70* | Stress | Sigma factors | Proteobacteria | Sulfitobacter |
|  |  | 13 | 134134270 | 3 | 42 | *narH* | Stress | Oxygen limitation | Proteobacteria | Burkholderia |
|  |  | 14 | 163839598 | 3 | 42 | *katE* | Stress | Oxygen stress | Actinobacteria | Renibacterium |
|  |  | 15 | 158435721 | 1 | 39 | *sigma_70* | Stress | Sigma factors | Firmicutes | Clostridium |
|  |  | 16 | 149937763 | 1 | 37 | *cda* | Carbon cycling | Carbon degradation | Bacteroidetes | Parabacteroides |
|  |  | 17 | 228848109 | 1 | 36 | *fnr* | Stress | Oxygen stress | Firmicutes | Bacillus |
|  |  | 18 | 225046340 | 4 | 18 | *cellobiase_bact_arch* | Carbon cycling | Carbon degradation | Firmicutes | Clostridium |
|  |  | 19 | 372474104 | 1 | 14 | *cellobiase_bact_arch* | Carbon cycling | Carbon degradation | Verrucomicrobia | Unclassified bacteria |
|  |  | 20 | 110833027 | 1 | 14 | *phoB* | Stress | Phosphate limitation | Proteobacteria | Alcanivorax |
|  |  | 21 | 13872764 | 1 | 14 | *acetylglucosaminidase_bact_arch* | Carbon cycling | Carbon degradation | Actinobacteria | Streptomyces |
|  | Connectors | 1 | 319924183 | 1 | 8 | *chitinase_general_bact_arch* | Carbon cycling | Carbon degradation | Acidobacteria | Terriglobus |
|  |  | 2 | 225175258 | 4 | 4 | *clpC* | Stress | Protein stress | Firmicutes | Dethiobacter |

**Table S4**. The abbreviation of phylogenetic taxa detected in this study

| Taxa | Code |  | Taxa | Code |
| --- | --- | --- | --- | --- |
| Actinobacteria | Ac |  | Firmicutes | Fm |
| Alphaproteobacteria | Ap |  | Fusobacteria | Fu |
| Aquificae | Aq |  | Gemmatimonadetes | Gm |
| Bacteroidetes | Ba |  | Gammaproteobacteria | Gp |
| Betaproteobacteria | Bp |  | Korarchaeota | Ko |
| Caldithrix | Ca |  | Lentisphaerae | Ls |
| Chlamydiae | Ch |  | Nitrospirae | Ns |
| Chlorobi | Cl |  | Planctomycetes | Pm |
| Chloroflexi | Co |  | Poribacteria | Pr |
| Crenarchaeota | Cr |  | Spirochaetes | Sc |
| Cyanobacteria | Cy |  | Synergistetes | Sy |
| Deferribacteres | Df |  | Thaumarchaeota | Ta |
| Deltaproteobacteria | Dp |  | Thermobaculum | Tb |
| Deinococcus-Thermus | DT |  | Thermodesulfobacteria | Td |
| Dictyoglomi | Dy |  | Tenericutes | Te |
| Elusimicrobia | El |  | Thermotogae | Tt |
| Epsilonproteobacteria | Ep |  | Uncultured archaea | Ua |
| Euryarchaeota | Eu |  | unclassified bacteria | Ub |
| Fibrobacteres | Fb |  | Uncultured euryarchaeote | Ue |
| Fungi | Fg |  | Unclassified proteobacteria | Up |


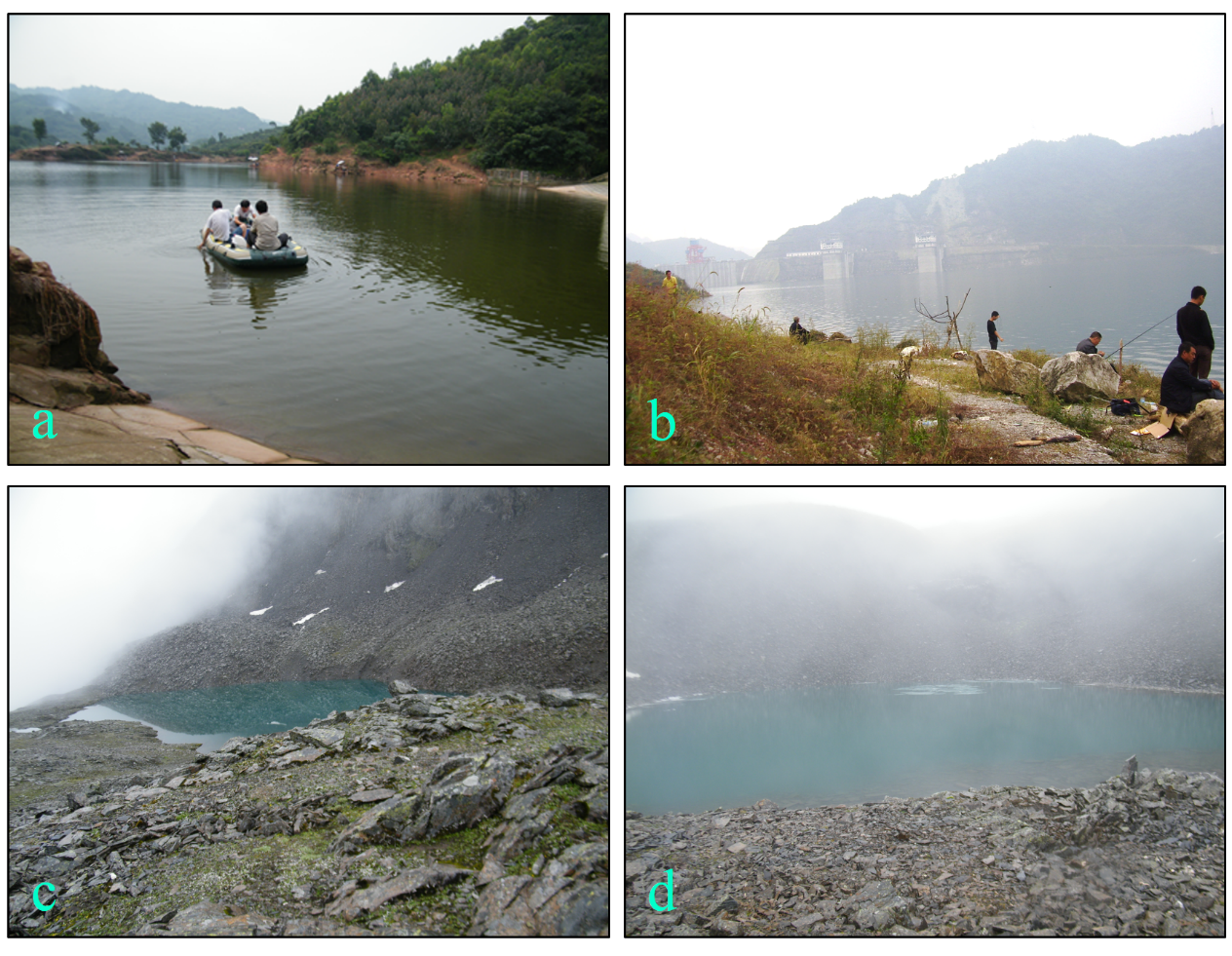


**Figure S1** Pictures of the investigated LELs (a: 525 m; b: 530 m) and HELs (c: 4,608 m; d: 4,652 m).


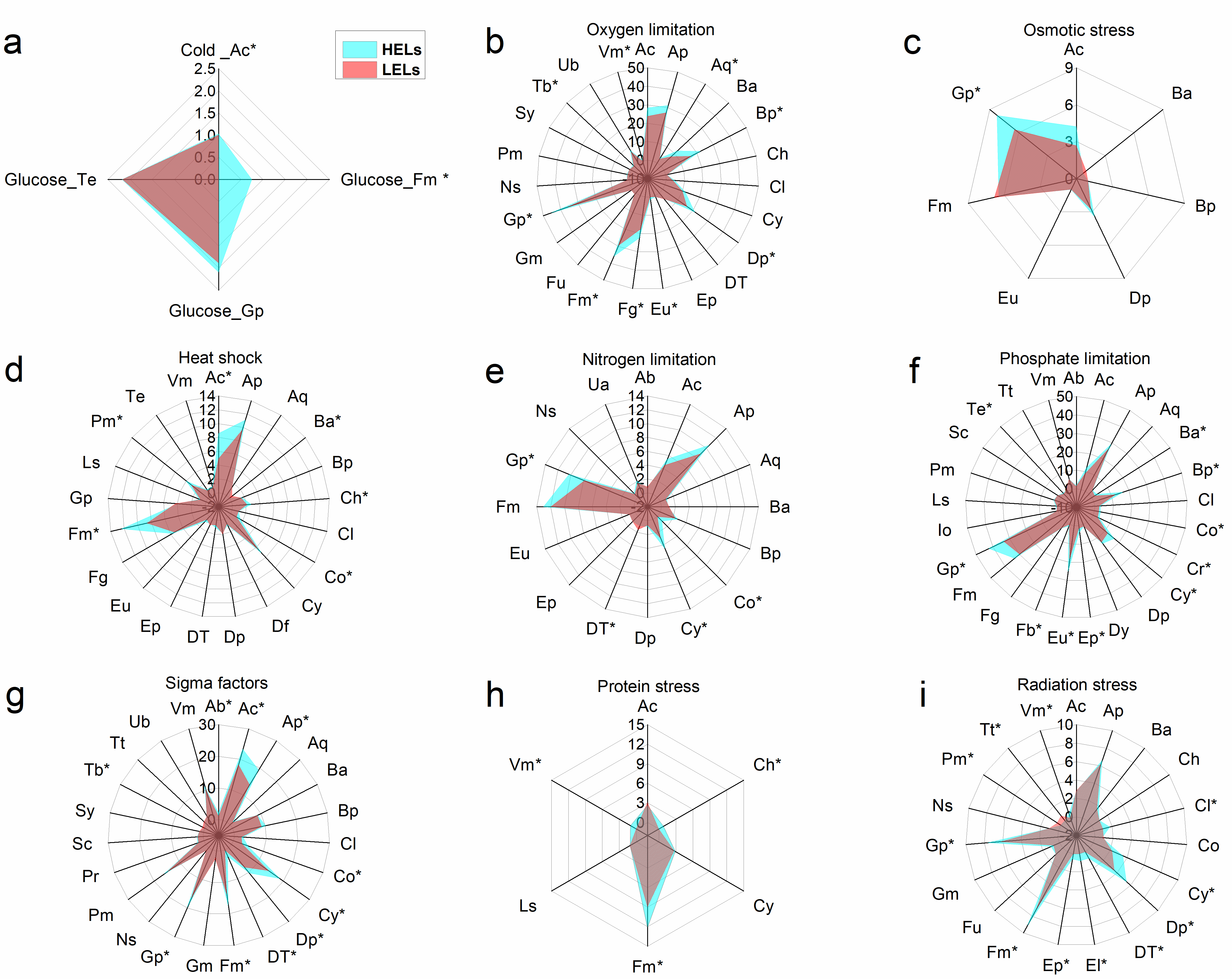


**Figure S2** Taxa–function relationships for stress response genes with significantly differences in normalized signal intensity between the HELs and the LELs. Mean values of samples from each lake were plotted for the genes represent (a) cold shock and glucose limitation, which were abbreviated as cold and glucose, respectively, in the figure, (b) heat shock, (c) nitrogen limitation, (d) osmotic stress, (e) oxygen stress, (f) phosphate limitation, (g) protein stress, (h) radiation stress, and (i) sigma factors. The two-character abbreviation of phylogenetic taxa was shown in Table S4. Asterisks (*) above the abbreviation of the taxa indicated significant differences (*p* < 0.05).


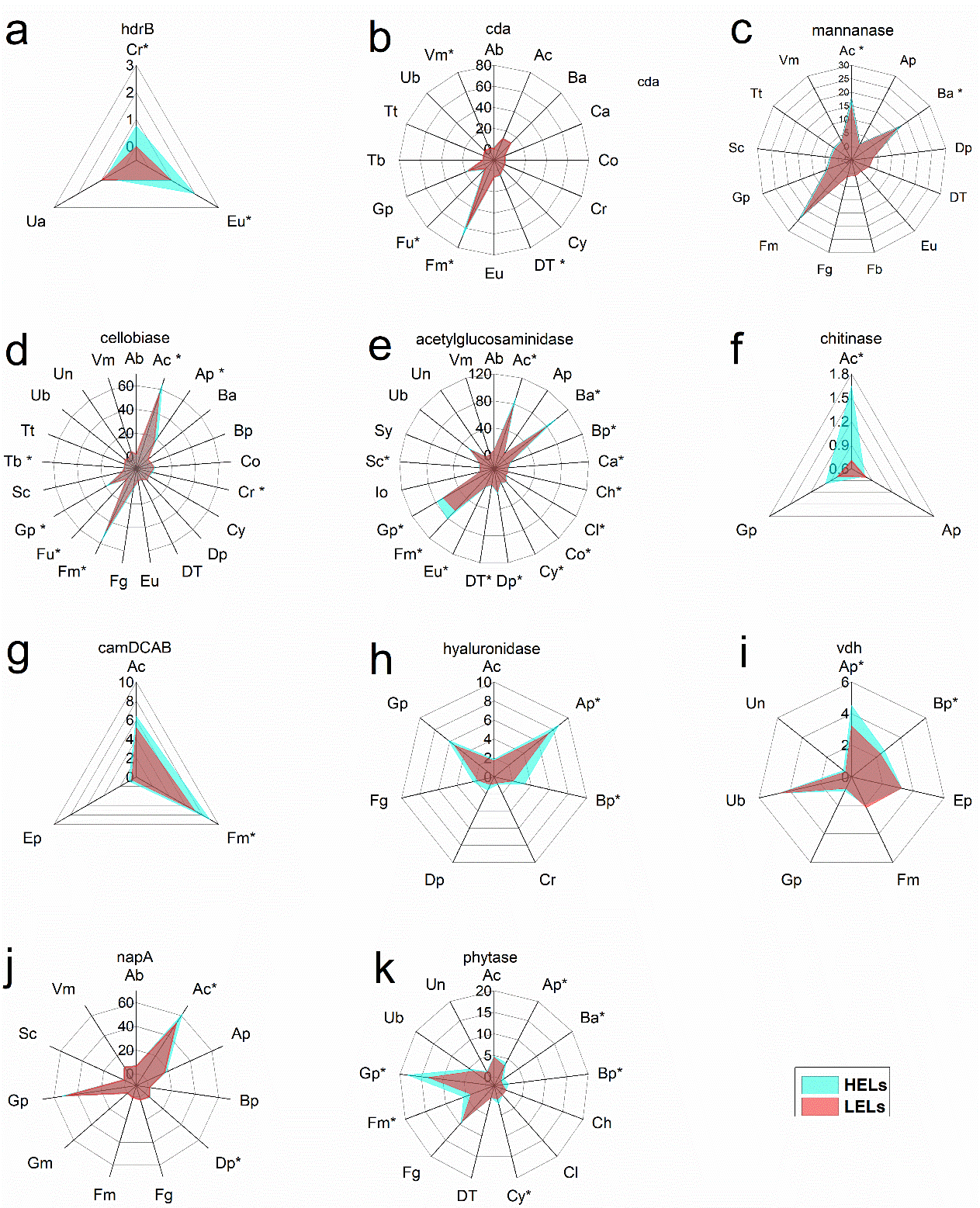


**Figure S3** Taxa–function relationships for carbon, nitrogen, and phosphorus cycling with significantly differences in normalized signal intensity between the HELs and the LELs. Mean values of samples from each lake were plotted for the genes represent (a) hdrB for Methane oxidation; (b) cda, (c) mannanase, (d) cellobiase, (e) acetylglucosaminidase, (f) chitinase, (g) camDCAB, and (h) hyaluronidase for carbon degradation; (j) napA for nitrate reduction and (k) phytase for phosphorus cycling. The two-character abbreviation of phylogenetic taxa was shown in Table S4. Asterisks (*) above the abbreviation of the taxa indicated significant differences (*p* < 0.05).

**
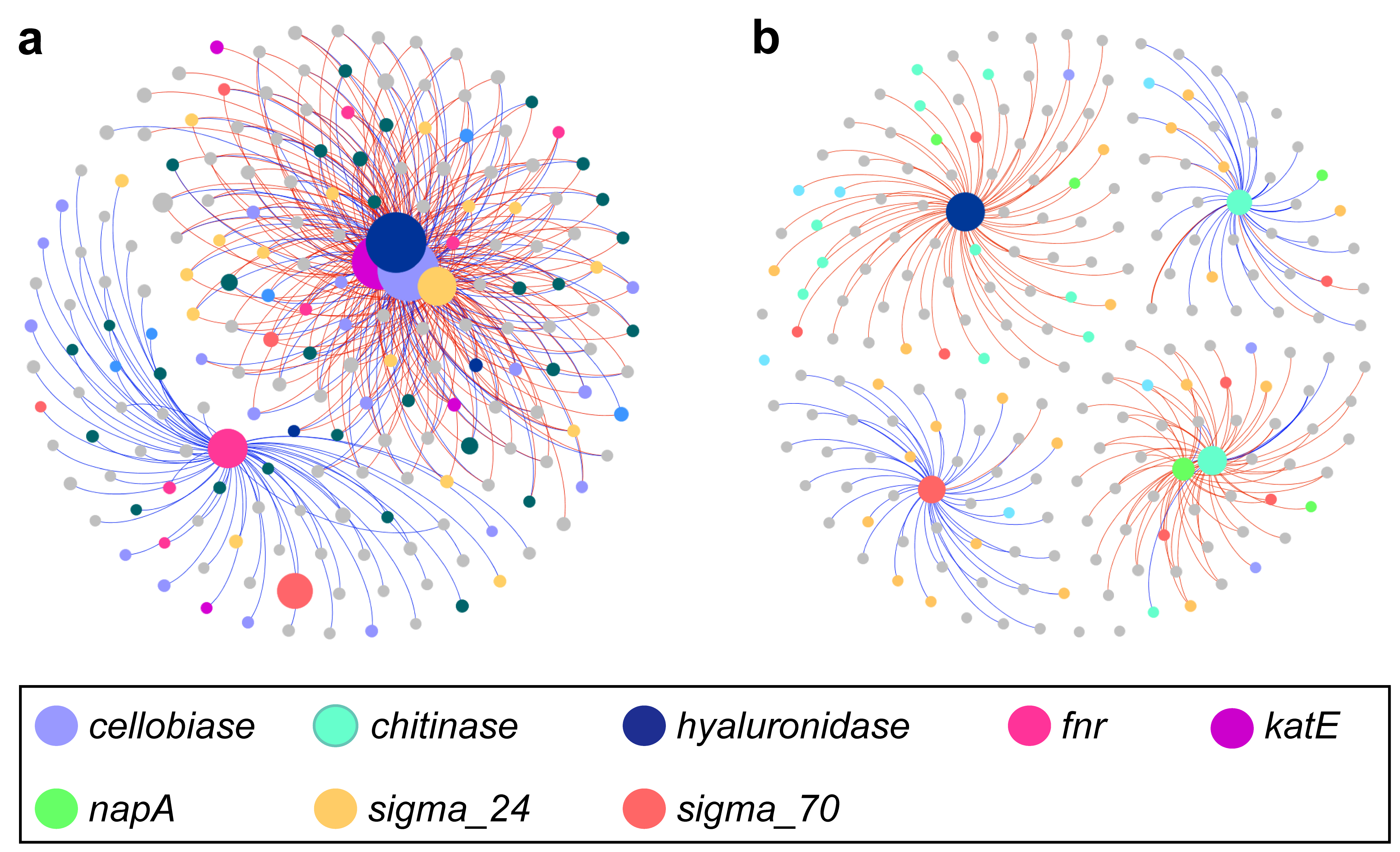
**

**Figure S4** Differences in the network interactions and the hub nodes of the microbial functional genes in the HELs (a) and LELs (b). Different colors of the nodes indicate different categories of functional genes. The lines connecting two nodes indicate interactions between different genes. The hub nodes in the top 1-5 modules were indicated by larger size. The detailed information of the large-sized hub nodes was shown in Table S3.

**
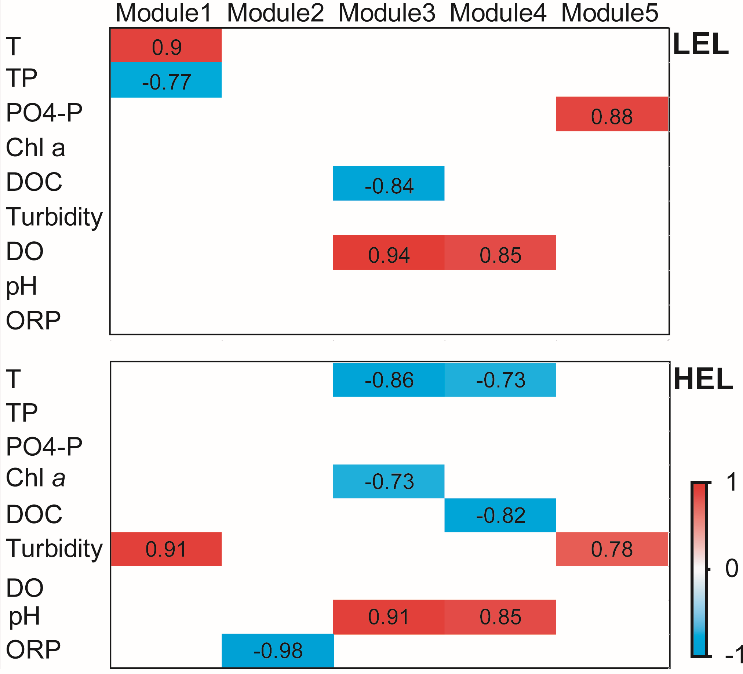
**

**Figure S5** Spearman's correlations between the environmental variables and the top 5 modules in functional gene networks of the HELs and the LELs. Only significant correlations (*p* < 0.05) are shown. T, water temperature; TP, total phosphorus; PO_4_-P, soluble phosphorus; Chl *a*, chlorophyll a; DOC, dissolved organic carbon; DO, dissolved oxygen; ORP, oxidation reduction potential.
